# Wearable Sensor-Based Human Activity Recognition: Performance and Interpretability of Dynamic Neural Networks

**DOI:** 10.3390/s25144420

**Published:** 2025-07-16

**Authors:** Dalius Navakauskas, Martynas Dumpis

**Affiliations:** Department of Electronic Systems, Vilnius Gediminas Technical University, Plytines g. 25-234, LT-10105 Vilnius, Lithuania; dalius.navakauskas@vilniustech.lt

**Keywords:** wearable sensors, human activity recognition, dynamic neural networks, LSTM, GRU, finite impulse response neural network, layer-wise relevance propagation

## Abstract

Human Activity Recognition (HAR) using wearable sensor data is increasingly important in healthcare, rehabilitation, and smart monitoring. This study systematically compared three dynamic neural network architectures—Finite Impulse Response Neural Network (FIRNN), Long Short-Term Memory (LSTM), and Gated Recurrent Unit (GRU)—to examine their suitability and specificity for HAR tasks. A controlled experimental setup was applied, training 16,500 models across different delay lengths and hidden neuron counts. The investigation focused on classification accuracy, computational cost, and model interpretability. LSTM achieved the highest classification accuracy (98.76%), followed by GRU (97.33%) and FIRNN (95.74%), with FIRNN offering the lowest computational complexity. To improve model transparency, Layer-wise Relevance Propagation (LRP) was applied to both input and hidden layers. The results showed that gyroscope Y-axis data was consistently the most informative, while accelerometer Y-axis data was the least informative. LRP analysis also revealed that GRU distributed relevance more broadly across hidden units, while FIRNN relied more on a small subset. These findings highlight trade-offs between performance, complexity, and interpretability and provide practical guidance for applying explainable neural wearable sensor-based HAR.

## 1. Introduction

Human Activity Recognition (HAR) based on wearable inertial measurement units, such as accelerometers and gyroscopes, has become an important topic in biomedical engineering and smart health systems. Accurate activity classification enables applications in rehabilitation, long-term monitoring, and fall prevention, especially among older adults and patients with mobility impairments [1]. Wearable sensors are compact and cost-effective and integrate easily into smartphones, smartwatches, and other mobile platforms, making them suitable for continuous, real-time monitoring in natural environments [2]. Advances in sensor integration have also produced multi-functional wearable devices capable of biometric identification and physiological signal monitoring, broadening their role in personalized health technologies [3].

Beyond general-purpose activity recognition, wearable HAR systems have found critical use in real-time fall detection and prevention, especially among elderly populations. Recent efforts have focused on pre-impact detection using embedded models to enable timely interventions, such as airbag activation. For instance, Turetta et al. [4] proposed a lightweight CNN optimized for real-time deployment on STM32 microcontrollers, demonstrating that deep models can operate effectively on constrained hardware. Wearable sensor systems have also been used in rehabilitation and sports performance monitoring, enabling continuous quantification of physical parameters and recovery progress [5]. These applications illustrate the broader role of HAR in personalized healthcare and movement analysis.

Recent HAR systems increasingly rely on dynamic neural networks (NNs), especially Long Short-Term Memory (LSTM), Gated Recurrent Unit (GRU), and Finite Impulse Response Neural Network (FIRNN) architectures, to model the temporal nature of sensor signals [6]. LSTM models, originally proposed by Hochreiter and Schmidhuber, are designed to capture long-range dependencies in sequential data and have been widely used in HAR research. GRUs offer similar accuracy but with fewer parameters, making them attractive for resource-constrained devices [7]. FIRNNs, although less popular, provide a simpler and interpretable structure and remain useful in real-time, embedded applications [8]. In this study, we focused specifically on dynamic neural networks that incorporate internal memory through recurrence or time-delayed filtering.

While dynamic NN-based HAR achieves high accuracy, a key limitation remains in understanding how these models make decisions. In clinical, wearable, and safety-critical settings, it is not enough to report accuracy alone; models must also offer explainability to ensure trust, diagnose failure modes, and guide sensor placement. Explainable artificial intelligence (XAI) methods such as Layer-wise Relevance Propagation (LRP) allow researchers to visualize which inputs or hidden units most influence model predictions [9,10]. However, many recent studies have focused mainly on explaining relevance at the input level or applied generic explanation techniques such as SHapley Additive exPlanations (SHAP) or Class Activation Maps (CAMs), without analyzing internal neuron contributions or comparing different temporal model types [11,12].

The main aim of this study was to systematically compare representative dynamic neural network architectures used for human activity recognition (HAR) tasks, in order to generate practical insights into their specificity and applicability when processing raw inertial sensor data. The object of investigation was a set of temporal models trained on raw inertial sensor data under controlled conditions. The investigation focused on three aspects: classification accuracy, computational cost, and model interpretability. Classification accuracy was evaluated by analyzing the impact of delay length and hidden neuron count, as well as confusion matrices. Computational cost was assessed through both theoretical complexity formulas and numeric metrics. Model interpretability was examined using layer-wise relevance propagation, with analyses performed at both the input and hidden-layer levels to reveal signal relevance patterns and internal activation behavior The activity set was deliberately constrained to support controlled analysis of model behavior rather than to maximize real-world classification coverage. This enabled clearer isolation of architectural effects on performance and explainability.

In summary, this study contributes a controlled and in-depth evaluation of the FIRNN, GRU, and LSTM architectures for sensor-based HAR. We highlight how model structure affects classification accuracy, computational demands, and interpretability using layer-wise relevance propagation. These insights aim to support researchers and engineers in selecting suitable architectures for real-time or safety-critical HAR scenarios. The remainder of this paper is structured as follows: Section 2 reviews recent advances in model-based HAR and explainability Section 3 outlines the dataset, preprocessing steps, and model configurations. Section 4 presents the experimental findings. Section 5 interprets their implications, and Section 6 summarizes the main takeaways.

## 2. Related Work

This section discusses recent studies related to HAR, emphasizing those that applied XAI methods to analyze sensor signals and NN models. Specifically, we review the methodologies used, significant findings, and how these studies have leveraged XAI to interpret their results. Moreover, we identify existing limitations and unexplored aspects that our research addresses—notably the, interpretability of internal NN states such as LSTM or GRU cell states and FIR filters in HAR applications.

### 2.1. Neural Network Architectures for HAR

NNs, particularly LSTM and GRU, have been widely adopted in HAR due their to efficiency in capturing sequential dependencies in sensor signals. Uddin and Soylu introduced a hybrid HAR approach combining LSTM with discriminant analysis, obtaining around 99% recall on public datasets using multiple wearable sensors [13]. Their model significantly improved HAR accuracy by effectively capturing sequential data dynamics but was evaluated primarily at the input-feature level rather than in deeper network states. Similarly, Raj and Kos employed convolutional neural networks (CNNs) on smartphone accelerometer data, achieving 97.2% classification accuracy under subject-aware conditions [14]. They noted that CNN models leveraged shorter signal segments to distinguish activities, emphasizing the local nature of CNN features, but did not explore recurrent or filter-level states within NNs.

In contrast, FIRNNs, though less common in recent literature, have been recognized for their inherent simplicity and interpretability advantages, which are especially relevant for real-time HAR applications. For instance, Wei and Wang utilized temporal convolutional networks, which are architecturally similar to FIRNNs, combined with attention mechanisms, achieving state-of-the-art performance (99%) and interpretability in intermediate layers, highlighting the practical value of FIR-like structures in HAR contexts [8].

Despite widespread adoption and notable successes, a comprehensive comparison and evaluation of these neural architectures in terms of both performance and internal interpretability remain underexplored, providing clear motivation for further investigation.

### 2.2. Explainable AI Methods in HAR

Explainability has become crucial in HAR research in terms of understanding model predictions better, enhancing trust in AI-driven decisions, and complying with regulatory standards, especially in critical biomedical contexts. Several recent studies have explored the use of XAI methods to interpret NN models trained on wearable sensor data.

Benos et al. developed an explainable HAR system for human–robot collaboration in agricultural settings [15]. They used SHAP with XGBoost classifiers to interpret accelerometer and gyroscope signals collected from multiple inertial sensors on different body parts. Their findings emphasized the lumbar, cervical, and chest sensors as the most critical for activity classification, providing practical guidance for sensor placement in real-world applications. However, their study mainly focused on feature importance at the input level, without exploring the internal NN representations, leaving room for further investigation into internal NN relevance and comparison of different neural architectures.

Similarly, Martins et al. explored posture recognition in occupational contexts (agriculture and construction) using hybrid CNN-LSTM and CNN-Transformer architectures with inertial sensors [16]. They applied Gradient-weighted Class Activation Mapping (Grad-CAM) to interpret CNN outputs, identifying the most influential sensor locations and signal segments. Their hybrid CNN-Transformer achieved an impressive 94.33% F1 score, highlighting CNN’s suitability for the extraction of discriminative features. However, while intermediate CNN representations were analyzed, they did not investigate recurrent cell states (LSTM or GRU), limiting the interpretability to convolutional layers only.

Mekruksavanich and Jitpattanakul applied Grad-CAM to a hybrid ConvNet–GRU model for HAR, visualizing which temporal segments and sensor axes influenced decisions [17]. Their subsequent study extended this to varying sensor positions using an attention-residual GRUs [18]. These contributions improved input-level interpretability but did not address the contributions of hidden neurons.

Pellano et al. studied XAI techniques in skeleton-based HAR using spatiotemporal graph CNNs [12]. By comparing CAM and Grad-CAM, they assessed which joints and time steps influenced classification. However, their work focused on skeletal inputs and graph networks, not inertial sensor signals or dynamic recurrent architectures.

Alves et al. generated t-distributed Stochastic Neighbor Embedding (t-SNE) visualizations of latent feature embeddings from 1D CNN models, offering insight into activity separation in feature space. This method highlighted internal structure but was not applied to temporal dynamic NNs [19]. The LIME model was applied to interpret electroencephalogram-based HAR models, identifying the spectral bands most responsible for activity classification. However, LIME remains architecture-agnostic and does not provide insight into sequential memory or hidden layer dynamics.

Bragança et al. demonstrated how SHAP can expose biases caused by different validation schemes in HAR [11] By comparing subject-wise and random splits, they showed how feature importance varied significantly, revealing overfitting to user-specific patterns. However, the analysis remained focused on feature-level explanations and did not extend into temporal dynamics.

Creagh et al. applied LRP to deep CNNs trained on smartphone-based accelerometer data to distinguish gait patterns of individuals with multiple sclerosis [10]. Their results demonstrated that LRP provided insights into gait speed, cadence-based measures, and perturbations in ambulatory signals relevant to MS-related impairments. Despite successfully using LRP to explain predictions, their study did not explicitly analyze internal NN states such as recurrent units or FIR filters, leaving a notable gap in the interpretability of internal neural dynamics.

In a broader context, Kaseris et al. presented a comprehensive survey on deep learning methods in HAR, emphasizing the increasing need for explainability. They highlighted that, while most current XAI efforts focus on input-level feature interpretation, there is insufficient exploration of internal layer states, particularly in recurrent architectures. This review underscored the demand for deeper interpretability in dynamic NNs such as LSTM and GRU architectures, which is precisely the gap our research addresses [20].

While existing XAI efforts have improved input-level understanding in HAR models, limited attention has been devoted to internal state relevance in dynamic NNs. Most studies have focused on CNNs or short-term signal segments, with few exploring neuron-wise contributions in temporal models such as LSTM, GRU, or FIRNN architectures. Additionally, few works offer comparative explainability across architectures. The present study addresses this gap by applying LRP to both input channels and hidden layers across FIRNN, LSTM, and GRU models trained on raw accelerometer and gyroscope data. This dual-level attribution approach yields new insights into how these neural networks represent and distribute information temporally, providing a deeper understanding of interpretability in HAR.

## 3. Materials and Methods

This section describes the dataset, NN architectures, training procedures, and explainability approach used in this study. Raw inertial data from wearable sensors were classified using three types of dynamic NNs—FIRNN, LSTM, and GRU—and interpreted using LRP to reveal input-level and hidden-layer contributions to each prediction.

### 3.1. Dataset Description

The Korean University HAR dataset [21], which is openly available in the Kaggle and Mendeley data repositories, was employed in this study. This dataset consists of accelerometer (m/$s^{2}$) and gyroscope (rad/s) signals collected from smartphones at a sampling rate of 100 Hz. It comprises 18 different activities performed by 90 subjects (75 males and 15 females), with a total of 1945 raw samples 20,750 three-second subsamples extracted from these raw samples. Each subsample contains 1800 data points (300 points for each of the three axes for both accelerometer and gyroscope signals).

In this study, only three activities were selected: sit-up, walk, and stairs up. These activities were chosen because of their distinct and repetitive motion characteristics, simplifying the classification task and providing clearer conditions for interpreting NN predictions via XAI techniques. Raw sensor data from the selected activities were directly utilized without manual feature extraction, preserving intrinsic temporal dynamics crucial for neural network architectures with memory capabilities.

During preprocessing, rows containing any zero values across the six sensor channels were excluded to eliminate corrupted or disconnected segments. Subsequently, class balancing was performed by truncating the number of samples per activity to ensure equal representation across the three selected classes. The data were randomly partitioned into training (70%), validation (15%), and testing (15%) subsets. To standardize the dataset for effective NN training, standard score normalization (Z-score) was applied separately for each activity class. Z-score normalization transforms each raw data point (*x*) into a standardized value ($x_{\mathrm{norm}}$) as follows:

(1) $$x_{i,\mathrm{norm}}\left(n\right)=\frac{x_{i}\left(n\right)-\mu_{i}}{\sigma_{i}},$$

where $x_{i}\left(n\right)$ is the original data point from the *i*-th sensor channel at time *n*, $\mu_{i}$ is the mean, and $\sigma_{i}$ is the standard deviation computed from all data points within the corresponding class and sensor channel [22]. This normalization procedure ensures data is centered around zero with unit variance, facilitating stable and efficient NN training [23].

### 3.2. Considered Dynamic Neural Networks

Three dynamic NN architectures were selected to analyze HAR from wearable sensor data: FIRNN, LSTM, and GRU. The selection motivated by their effectiveness in modeling temporal dependencies in sequential sensor data, which is crucial for accurate activity classification. These architectures also maintain full time-step granularity throughout the network, making them particularly suitable for consistent analysis of temporal relevance and memory behavior. In contrast, traditional classifiers such as SVM or RF typically rely on precomputed statistical features and lack explicit memory structures, making them less appropriate for analyzing temporal modeling behavior from raw signals. Similarly, CNN-based HAR models often involve temporal pooling or strides, which reduce time-step resolution and complicate interpretability at the level of individual time points. While powerful sequence models like Transformers offer strong performance, their significantly larger computational demands and architectural complexity—along with the need for additional assumptions for fair comparison—render them unsuitable for the controlled evaluation framework applied in this study.

#### 3.2.1. Finite Impulse Response Neural Network

The FIRNN was selected for its explicit temporal modeling capabilities and inherent interpretability due to its linear filtering structure. Each hidden neuron employs finite impulse response filters as synapses. The recall for the *h*-th hidden neuron is computed as follows:

(2) $$x_{h}^{\left(1\right)}\left(n\right)=\Psi_{S}\left(\sum_{i=1}^{N_{I}}\sum_{j=1}^{N_{D}}w_{ijh}^{\left(1\right)}x^{\left(0\right)}\left(n-j-i\right)-{\bar{w}}_{h}^{\left(1\right)}\right),$$

where $W^{\left(1\right)}\in R^{N_{I}\times N_{D}\times N_{H}}$ is the FIR filter weight tensor and ${\bar{w}}^{\left(1\right)}\in R^{N_{H}}$ is the bias vector.

#### 3.2.2. Long Short-Term Memory

LSTM, introduced by Hochreiter and Schmidhuber [24], offers and advanced memory-gating mechanisms to capture long-range temporal dependencies effectively.

The recall of the *h*-th hidden neuron is described as follows:

(3) $$\begin{array}{cc} x_{h}^{\left(1\right)}\left(n\right) & =x_{Oh}^{\left(1\right)}\left(n\right)\Psi_{T}\left(x_{Ch}^{\left(1\right)}\left(n\right)\right), \end{array}$$

(4) $$\begin{array}{cc} x_{Ch}^{\left(1\right)}\left(n\right) & =x_{Fh}^{\left(1\right)}\left(n\right)x_{Ch}^{\left(1\right)}\left(n-1\right)+x_{Ih}^{\left(1\right)}\left(n\right){\tilde{x}}_{Ch}^{\left(1\right)}\left(n\right), \end{array}$$

(5) $$\begin{array}{cc} {\tilde{x}}_{Ch}^{\left(1\right)}\left(n\right) & =\Psi_{T}\left(\sum_{i=1}^{N_{I}}\sum_{j=1}^{N_{D}}w_{Cijh}^{\left(1\right)}\left(x^{\left(0\right)}\left(n-i-1\right)+x_{h}^{\left(1\right)}\left(n-j\right)\right)-{\bar{w_{C}}}_{h}^{\left(1\right)}\right), \end{array}$$

(6) $$\begin{array}{cc} x_{Ih}^{\left(1\right)}\left(n\right) & =\Psi_{S}\left(\sum_{i=1}^{N_{I}}\sum_{j=1}^{N_{D}}w_{Iijh}^{\left(1\right)}\left(x^{\left(0\right)}\left(n-i-1\right)+x_{h}^{\left(1\right)}\left(n-j\right)\right)-{\bar{w}}_{Ih}^{\left(1\right)}\right), \end{array}$$

where the input, forget, and output gates and the cell candidate are parameterized by weight tensors $W_{I}^{\left(1\right)},W_{F}^{\left(1\right)},W_{O}^{\left(1\right)},W_{C}^{\left(1\right)}\in R^{N_{I}\times N_{D}\times N_{H}}$ and bias vectors ${\bar{w}}_{I}^{\left(1\right)},{\bar{w}}_{F}^{\left(1\right)},{\bar{w}}_{O}^{\left(1\right)},{\bar{w}}_{C}^{\left(1\right)}\in R^{N_{H}}$.

#### 3.2.3. Gated Recurrent Unit

The GRU architecture, introduced by Cho et al. [25], provides efficient modeling of sequential data with a simplified gating mechanism compared to LSTM, reducing computational complexity. The hidden neuron recall ($s_{h}^{\left(1\right)}\left(n\right)$) is computed as follows:

(7) $$\begin{array}{cc} x_{h}^{\left(1\right)}\left(n\right) & =x_{Uh}^{\left(1\right)}\left(n\right)x_{h}^{\left(1\right)}\left(n-1\right)+\left(1-x_{Uh}^{\left(1\right)}\left(n\right)\right){\tilde{x}}_{h}^{\left(1\right)}\left(n\right), \end{array}$$

(8) $$\begin{array}{cc} {\tilde{x}}_{h}^{\left(1\right)}\left(n\right) & =\Psi_{T}\left(\sum_{i=1}^{N_{I}}\sum_{j=1}^{N_{D}}w_{ijh}^{\left(1\right)}\left(x^{\left(0\right)}\left(n-i-1\right)+x_{h}^{\left(1\right)}\left(n-j\right)x_{Rh}^{\left(1\right)}\left(n\right)\right)-{\bar{w}}_{h}^{\left(1\right)}\right), \end{array}$$

(9) $$\begin{array}{cc} x_{Rh}^{\left(1\right)}\left(n\right) & =\Psi_{S}\left(\sum_{i=1}^{N_{I}}\sum_{j=1}^{N_{D}}w_{Rijh}^{\left(1\right)}\left(x^{\left(0\right)}\left(n-i-1\right)+x_{h}^{\left(1\right)}\left(n-j\right)\right)-{\bar{w}}_{Rh}^{\left(1\right)}\right), \end{array}$$

(10) $$\begin{array}{cc} x_{Uh}^{\left(1\right)}\left(n\right) & =\Psi_{S}\left(\sum_{i=1}^{N_{I}}\sum_{j=1}^{N_{D}}w_{Uijh}^{\left(1\right)}\left(x^{\left(0\right)}\left(n-i-1\right)+x_{h}^{\left(1\right)}\left(n-j\right)\right)-{\bar{w}}_{Uh}^{\left(1\right)}\right), \end{array}$$

where the update, reset, and candidate states each use weight tensors ($W_{U}^{\left(1\right)},W_{R}^{\left(1\right)},W^{\left(1\right)}\in R^{N_{I}\times N_{D}\times N_{H}}$) with corresponding bias vectors (${\bar{w}}_{U}^{\left(1\right)},{\bar{w}}_{R}^{\left(1\right)},{\bar{w}}^{\left(1\right)}\in R^{N_{H}}$).

All three NN architectures utilize logistic sigmoid and hyperbolic tangent functions due to their proven effectiveness in introducing non-linearity and gating functionalities crucial for dynamic modeling of sequential data [26]. Output-layer activations employ softmax functions to produce class probabilities [23].

### 3.3. Investigation Procedure

The experimental procedure consisted of systematically evaluating the dynamic NNs (FIRNN, LSTM, and GRU) across varying structural parameters. Each NN was initialized with weights drawn from a scaled normal distribution (N(0,0.001)), facilitating stable training conditions and avoiding premature activation saturation [27]. Logistic sigmoid ($\Psi_{S}$) and hyperbolic tangent ($\Psi_{T}$) activation functions were utilized in hidden layers, while the softmax function was applied in the output layer to generate class probabilities [23].

The experiments explored two main hyperparameters: NN delays and hidden neuron counts. Delays, representing the length of input signal windows, were systematically varied from 1 to 100 samples in increments of 10, enabling an extensive evaluation of the impact of temporal context on recognition accuracy. Hidden neuron counts were varied among five discrete levels (2, 4, 6, 8, and 10), allowing for analysis of the relationship between NN complexity and performance.

Each unique combination of these hyperparameters underwent 100 independent training repetitions, each initialized with unique random weights. This repetitive training approach was employed to ensure statistically robust and consistent results, accounting for variability due to random initialization. In total, 3 NN architectures, 11 delay configurations, 5 hidden neuron configurations, and 100 repetitions per configuration resulted in 16,500 distinct NN training cycles. Networks demonstrating the highest validation accuracy were selected for subsequent interpretability analyses.

### 3.4. Explainability Metric

To interpret the model decisions, LRP was applied to compute relevance scores for both input channels and internal hidden units [28]. LRP assigns a scalar relevance score to each neuron or input feature by redistributing the prediction backward through the NN, preserving total relevance in each layer.

The standard LRP rule used in this study is expressed as follows:

(11) $$R_{i}^{\left(l\right)}=\sum_{j}\frac{x_{i}^{\left(l\right)}w_{ij}^{\left(l,l+1\right)}}{\sum_{i^{\prime }}x_{i^{\prime }}^{\left(l\right)}w_{i^{\prime }j}^{\left(l,l+1\right)}+\epsilon }R_{j}^{\left(l+1\right)},$$

where $R_{i}^{\left(l\right)}$ and $R_{j}^{\left(l+1\right)}$ are the relevance scores of neuron *i* in layer *l* and neuron *j* in layer l+1, $x_{i}^{\left(l\right)}$ is the post-activation output of neuron *i* in layer *l*, $w_{ij}^{\left(l,l+1\right)}$ is the weight connecting neuron *i* in layer *l* to neuron *j* in layer l+1, $i^{\prime }$ is a dummy index running over *all* neurons in layer *l* (so the denominator’s sum ($\sum_{i^{\prime }}$) collects contributions from every neuron ($i^{\prime }$) in the same layer as *i*), and $\epsilon =10^{-6}$ is a small stabilization term.

Architecture-specific adaptations were implemented. In FIRNN, LRP was propagated through the FIR filters. For GRU, the gating structure (reset and update gates) was explicitly modeled, ensuring that the relevance flow respected temporal memory. In LSTM, relevance propagation was adapted to account for input, forget, and output gates, as well as cell-state contributions [29,30].

Proportional normalization was consistently applied to relevance scores for both temporal and global analyses. For temporal analysis, channel-wise normalized relevance scores at each time step were computed as follows:

(12) $$T_{h}\left(n\right)=\frac{\left|R_{h}\left(n\right)\right|}{\sum_{c=1}^{N_{C}}\sum_{t=1}^{N_{T}}\left|R_{c}\left(t\right)\right|},$$

where $R_{h}\left(n\right)$ is the relevance of channel or hidden neuron *h* at time step *n*, $N_{C}$ is the total number of channels/hidden units, and $N_{T}$ is the the length of the temporal window.

For global analysis, the normalized relevance was averaged across all time steps in a window, yielding the relative global relevance per channel or neuron:

(13) $$V_{h}=\frac{\sum_{n=1}^{N_{T}}\left|R_{h}\left(n\right)\right|}{\sum_{c=1}^{N_{C}}\sum_{n=1}^{N_{T}}\left|R_{c}\left(n\right)\right|}.$$

Normalized relevance maps were derived from all test samples, averaged class-wise, and computed using the best-performing NN instances (selected by validation accuracy). Consistent normalization facilitates clear comparisons across time segments, NN architectures, and classes, avoiding biases due to absolute magnitude differences [15,31].

## 4. Results

This section presents the classification performance, computational complexity, and explainability analysis of the evaluated dynamic neural networks. The results include accuracy comparisons, confusion matrix evaluation, input relevance distributions, and hidden-layer relevance derived using LRP.

### 4.1. Neural Network Performance

In order to evaluate the dynamic NN architectures from both computational and accuracy perspectives in detail, first, computational complexity formulas were derived and analyzed. Then, these complexity metrics were quantified for the best-performing configurations of each NN to investigate the trade-offs between classification accuracy and resource requirements. Finally, the effect of varying hyperparameters, such as the delay length and number of hidden neurons, was analyzed to understand how temporal context and NN capacity influence recognition accuracy across different architectures.

#### 4.1.1. Computational Complexity of Neural Network Architectures

Table 1 summarizes computational complexity for FIRNN, GRU, and LSTM. It is expressed through the total number of binary additions ($N_{2\Sigma }$), binary multiplications ($N_{2\Pi }$), activation functions ($N_{\Psi }$), and total weights ($N_{W}$). Gray color indicates a minimum value.

The provided complexity formulas illustrate distinct computational demands across the three NN types. FIRNNs have the simplest structure, with linear dependence on the number of inputs ($N_{I}$), delays ($N_{D}$), and hidden units ($N_{H}$). They require the fewest binary operations and activation functions, making them well-suited for low-power or real-time applications.

GRU and LSTM introduce significantly higher complexity due to their gating mechanisms. GRUs require approximately three times more additions and multiplications than FIRNN for the same delay and hidden size, while LSTM models require about four times the addition operations and the largest number of activation functions ($N_{\Psi }$). LSTM models also have the biggest number of trainable parameters ($N_{W}$) because each unit uses four gates, while GRUs use three.

Overall, FIRNN is the least computationally demanding, while LSTM incurs the highest complexity cost. GRU sits between the two in complexity, providing a trade-off between resource requirements and modeling capacity. This analysis is critical for choosing an NN architecture in scenarios constrained by computation or memory, such as on-device or embedded HAR applications.

#### 4.1.2. Highest Accuracy Neural Networks and Their Computational Complexity

Numeric complexity values for FIRNN, GRU, and LSTM at their highest achieved classification accuracy are presented in Table 2. All NNs achieved peak accuracy under an identical $N_{D}=100$, $N_{H}=10$, $N_{I}=6$, and $N_{C}=3$. Gray color outlines a minimum values.

The results confirm that LSTM achieved the highest accuracy (98.76%), albeit at significantly higher computational complexity compared to GRU and FIRNNs. GRU provides a balanced trade-off between accuracy (97.33%) and computational requirements. FIRNNs achieved the lowest accuracy (95.74%) but minimal computational complexity, showing their suitability for embedded real-time applications.

#### 4.1.3. Accuracy Variation with Network Hyperparameters

**Figure 1:**
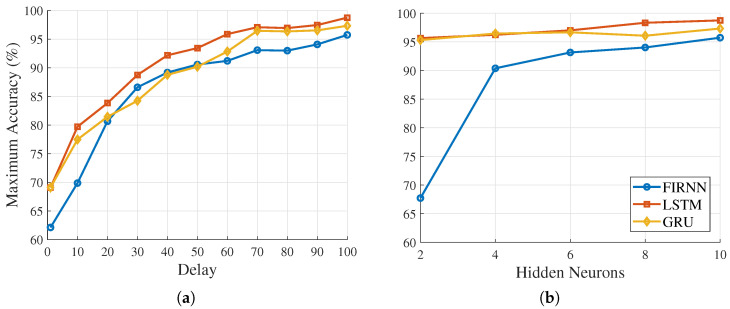
FIRNN, LSTM, and GRU classification accuracy: (**a**) dependency on the number of delays; (**b**) dependency on the number of hidden neurons. Each point represents the highest achieved accuracy across 100 different experimental runs.

The influence of delay length and the number of hidden neurons on classification accuracy for FIRNN, LSTM, and GRU is presented in Figure 1 (see on the next page).

From Figure 1a, it is clear that increasing delays significantly improves the classification accuracy for all three NN architectures. Specifically, accuracy increases from 69.10% to 98.76% for LSTM, from 69.10% to 97.33% for GRU, and from 62.13% to 95.73% for FIRNN when the number of delays increases from 1 to 100. These results indicate that having a sufficient amount of past temporal information is essential to achieve high accuracy.

Figure 1b shows how accuracy changes with an increase in the number of hidden neurons. LSTM and GRU show moderate improvements in accuracy as the number of hidden neurons increases from 2 to 10 (from 95.66% to 98.76% for LSTM and from 95.32% to 97.33% for GRU). This suggests that these recurrent NNs perform well, even with a relatively small hidden-layer size. Further increases yield only marginal accuracy improvements.

The FIRNN, however, shows a stronger dependency on the number of hidden neurons, with a significant accuracy improvement from 67.73% (two neurons) to 90.39% (four neurons). Additional increases from 4 to 10 neurons result in relatively smaller improvements—from 90.39% to 95.73%. This indicates that FIRNNs need at least four hidden neurons for good classification accuracy, but beyond this, accuracy primarily increases with the number of delays rather than hidden-layer size.

The results clearly show the importance of delay length, or temporal context, for achieving high accuracy in all three NN architectures. For recurrent NNs (LSTM and GRU), a small number of hidden neurons already provides high accuracy. For FIRNNs, however, both the number of delays and a minimum hidden neuron count are critical.

### 4.2. Classification Error Analysis

**Figure 2:**
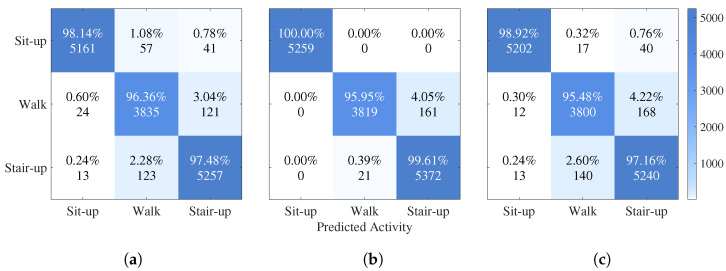
Confusion matrices for the (**a**) FIRNN, (**b**) LSTM, and (**c**) GRU models showing classification performance across sit-up, walking, and stair-climbing activities. Each panel represents one NN.

To analyze the detailed classification performance of the trained neural networks, confusion matrices were computed for the FIRNN, GRU, and LSTM models. Each confusion matrix visualizes the percentage and absolute number of samples classified correctly and incorrectly across the three activity classes: sit-up, walking, and stair climbing. Figure 2 presents the confusion matrices for the three NNs, side by side within a single combined figure for easier comparison.

For the FIRNN, sit-up activities were classified with 98.14% accuracy (Figure 2a). However, there was noticeable confusion between walking and stair-climbing activities, with 3.04% of walking samples being classified as stair climbing. Although the overall classification performance was strong, the FIRNN struggled to fully separate dynamic activities without recurrent structures.

The LSTM achieved perfect classification for the sit-up activity (100.00%), and stair climbing was recognized with 99.61% accuracy (Figure 2b). Only 4.05% of walking samples were misclassified as stair climbing. These results demonstrate that LSTMs, with their ability to capture long-term dependencies, provide superior discrimination between activities with similar temporal patterns.

The GRU also performed very well, achieving 98.92% accuracy for sit-up and 97.16% for stair climbing (Figure 2c). Walking activities showed a 95.48% classification accuracy, with 4.22% of samples misclassified as stair climbing, which is slightly worse compared to LSTM. This indicates that, while GRUs are effective at sequence modeling, they are somewhat less precise than LSTM for fine-grained activity separation.

In all NNs, walking was the most commonly misclassified activity, with occasional confusion against stair climbing—particularly in the FIRNN (3.04%) and GRU (4.22%) models. While the overall misclassification levels remain low and model performance remains strong under the given experimental conditions, this confusion likely stems from similarities in dynamic patterns: both activities involve periodic vertical acceleration patterns that are difficult to distinguish using a single sensor. Although not indicative of a major limitation, this observation suggests that robustness challenges may emerge in more complex real-world settings, where activity transitions and sensor variability are more likely to occur.

### 4.3. Explainability of Inputs

To understand how different NNs utilize input sensor signals for decision-making, explainability analyses were performed using LRP. This section presents results at the input level, showing how relevance is distributed across time and sensor channels. The goal is to interpret when and where each NN focuses its attention during activity recognition and how this attention relates to the underlying signal dynamics. Two types of analysis are included: temporal relevance maps, which show fine-grained patterns of input contribution over time, and a quadrant-based summary linking input relevance with signal variation (quantified via the zero-crossing rate). These complementary views provide insight into the temporal selectivity and modality preferences of FIR, LSTM, and GRU.

#### 4.3.1. Temporal Relevance Analysis

**Figure 3:**
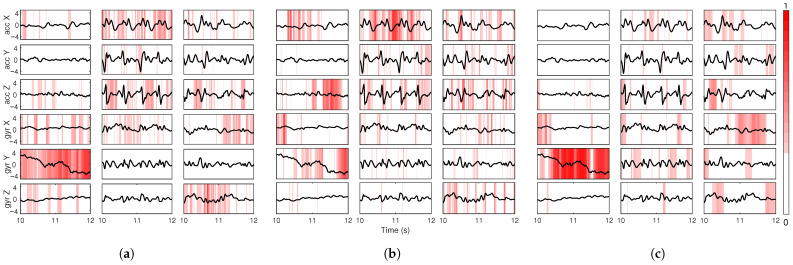
Example input LRP for (**a**) FIRNN, (**b**) LSTM, and (**c**) GRU across sit-up, walking, and stair-climbing classes. Each subplot shows a 2 s fragment of the raw sensor signal (black) and the corresponding LRP relevance (red color map) for one input channel. Darker red indicates higher relevance.

To explore how different input channels contribute over time, LRP relevance maps were plotted for each sensor axis across three representative activity sequences: sit-up, walking and stair climbing (Figure 3).

The figure show the raw input signals (black lines) overlaid with time-resolved LRP values using a red color scale. Each subplot represents one input channel, with darker red regions indicating higher relevance. These visualizations were generated without applying percentile clipping or sparsification, allowing for full expression of relevance magnitudes ranging from 0 to 1.

The results reveal clear differences in temporal importance patterns across NNs and classes. For the FIRNN, relevance appears more uniformly distributed in time but is concentrated in a small subset of channels, notably *gyr Y* during sit-ups and *gyr X* during walking. In contrast, the LSTM displays more pronounced, temporally localized relevance bursts in multiple channels, including *acc Z* and *gyr Z* for stair climbing. GRU shares a similar pattern but tends to focus on fewer channels per class, emphasizing *gyr Y* during sit-ups and *gyr X* during walking. Across all neural networks, the *acc Y* channel consistently shows lower relevance, suggesting limited utility in activity discrimination.

#### 4.3.2. Input Relevance vs. Zero-Crossing Rate

To explore the relationship between input dynamics and model attention, quadrant plots were constructed by comparing the zero-crossing rate (ZCR) with the input relevance. The ZCR captures the temporal variability of sensor signals and provides a lightweight descriptor of signal dynamics. While more sophisticated frequency-based explainability methods exist [32], the ZCR offers a practical and interpretable measure for highlighting differences in activity patterns. For relevance, the number of time points exceeding the top-30% relevance threshold was counted per input channel. This sparsification highlights the most informative signal segments and reduces the influence of low-relevance regions. Similar top-*k* filtering strategies have been used in recent explainability studies to enhance clarity and reduce noise in visualizations [15].

**Figure 4:**
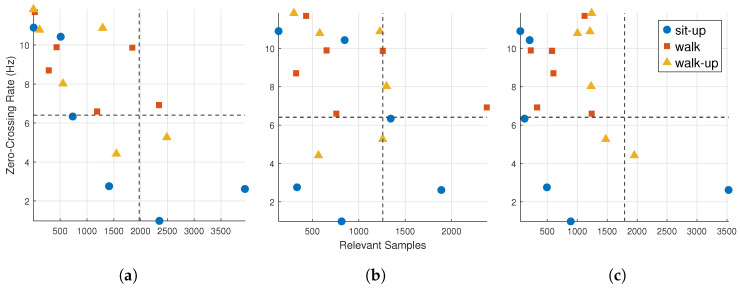
Quadrant plots of input relevance count vs. zero-crossing rate (ZCR) for (**a**) FIRNN, (**b**) LSTM, and (**c**) GRU. Each point corresponds to one input channel and activity class. Different marker shapes represent different classes: sit-up (circles), walking (squares), or stair climbing (triangles). Vertical and horizontal dashed lines mark median thresholds (ZCR = 6.3 Hz, relevance count = 1740).

Figure 4 displays quadrant plots for the FIRNN, LSTM, and GRU models, with each point representing one input channel for a specific activity.

This comparison highlights the relationship between signal dynamics and model attention, showing which inputs exhibit both high temporal variation and high relevance. FIRNN and LSTM display a wider spread across all quadrants, while GRU shows a more focused pattern. Specifically, FIRNN has 3 points each in Q1 and Q2 and 11 in Q4, suggesting mixed input dynamics, with a strong focus on high-relevance, high-ZCR signals. LSTM distributes four points in Q1, two in Q2, three in Q3, and nine in Q4, reflecting diverse temporal behavior. GRU concentrates heavily in Q4 (12 points), with only six inputs elsewhere, indicating a stronger bias toward inputs that are both dynamic and relevant.

To quantify the distribution, Table 3 summarizes the number of input channels per class falling into each quadrant. Each activity contributes six input points (one per sensor axis). Gray color indicates values to note.

The results reveal that, from a class-wise perspective, walking and stair climbing consistently show strong alignment with dynamic and relevant signals (Q2) across all NN types. Notably, all three NNs allocate four or more of the six input channels for these activities into Q2, indicating a shared dependence on temporally active sensor inputs for recognizing gait-based movements. In contrast, sit-ups show a more dispersed pattern, particularly for FIRNN and LSTM, which split their input channels evenly across Q1, Q3, and Q4. This suggests that sit-up recognition does not rely as strongly on dynamic input variation and, instead, leverages lower-frequency or less temporally active features. GRU shows the sharpest contrast across classes, allocating all six walk-related channels to Q2 but distributing sit-up inputs predominantly in Q3 and Q4. These findings reinforce the view that dynamic NNs adapt their attention not only based on architecture but also activity type, with cyclic movements like walking benefiting most from high-ZCR, high-relevance sensor inputs.

#### 4.3.3. Global Input Relevance

To understand which sensor channels each NN used most strongly, global input relevance was analyzed using LRP. The analysis was performed in two parts: first, by summing relevance across all activity classes to assess overall usage, then by separating per-class contributions to evaluate how sensor importance varies between different movements.

**Figure 5:**
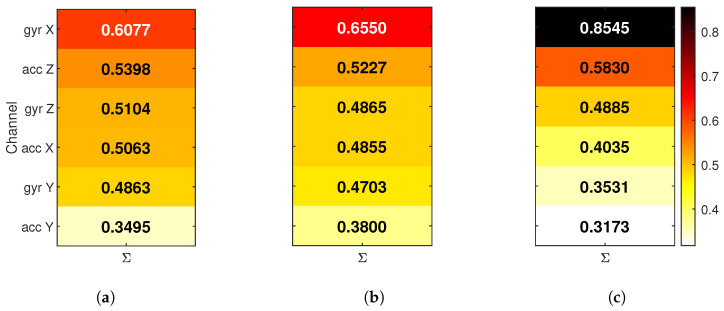
Accumulated channel relevance per model for (**a**) FIRNN, (**b**) LSTM, and (**c**) GRU. Each heat map shows the total normalized contribution of each input channel across all classes. Inputs are sorted by total importance.

In the first step, for each input channel, the absolute relevance values were accumulated, then normalized so that the total relevance per NN was comparable. The results are shown in Figure 5, where each bar reflects how much a particular input channel contributed to the model’s predictions overall. In this view, inputs are sorted by total relevance per NN, and the color scale is shared across models.

The accumulated relevance maps shows several key differences between models. The FIRNN uses a relatively balanced set of input channels, with five out of six contributing meaningfully and the *acc Y* receiving the least attention. LSTM shows a similar pattern, where importance is distributed more evenly across accelerometer and gyroscope inputs. In contrast, GRU places a very large portion of total relevance on a single input—*gyr Y*, which, alone, receives nearly 50% of the total attribution. This suggests that GRU relies more heavily on one or two dominant inputs, while FIRNN and LSTM combine information from a wider set of sensor axes.

**Figure 6:**
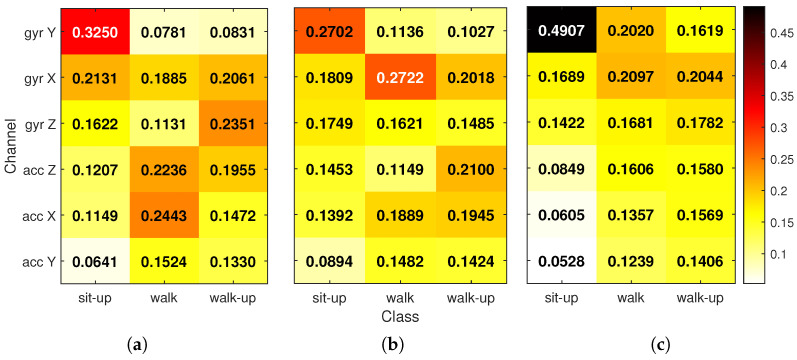
Class-conditional input relevance for (**a**) FIRNN, (**b**) LSTM, and (**c**) GRU based on LRP. Each heat map shows the relative contribution of each input channel to class predictions. For each column (activity), the six values sum to 1. Numerical ratios are shown in each cell.

To explore how relevance varies depending on the type of movement, a second view was created by computing class-specific relevance. For each activity class, LRP values were summed over time and normalized across the six input channels. This resulted in a per-class distribution of relevance that shows which sensors are most important for recognizing each activity. The final relevance matrices were then averaged across all correct predictions per class. Figure 6 shows the results, where each heatmap cell represents the proportion of relevance attributed to a given input for a specific class. The numbers overlaid in each cell make the differences easier to interpret.

From these class-specific heat maps, several additional insights emerge. For the sit-up activity, all three models rely most heavily on the *gyr Y*, which captures upper-body bending motion. GRU places almost half of the relevance on this single channel, while FIRNN and LSTM distribute it more broadly. For the walking and stair-climbing classes, input relevance is typically split across two to three sensor axes. These activities involve more complex movement, often requiring input from both accelerometers and gyroscopes to distinguish between motion patterns. LSTM, again, shows a more balanced relevance profile across inputs, while GRU tends to assign sharper peaks to one or two sensors. The FIRNN falls in between, using a mix of accelerometer and gyroscope channels, depending on the class.

Interestingly, across all models and activities, the *acc Y* consistently receives the lowest relevance values. This suggests that motion along this axis contributes the least to class separation. The class analysis also confirms that different NN architectures rely on input information in different ways: some extract decisions from a combination of multiple channels, while others focus more narrowly on dominant sources.

### 4.4. Explainability of Hidden Layers

To better understand how hidden units contribute to model decisions, relevance scores were analyzed using global LRP. This analysis was performed in two steps: first, by summing relevance values across all classes to obtain an overall view, then by examining class-specific contributions.

**Figure 7:**
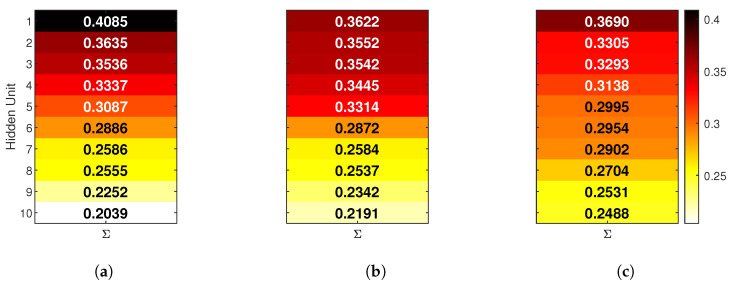
Accumulated hidden-unit relevance for (**a**) FIRNN, (**b**) LSTM, and (**c**) GRU networks. Each heat map shows the total normalized contribution of each hidden neuron summed over all classes. Units that, together, contribute more than 55% are highlighted with a black border.

The first view is shown in Figure 7, which presents the total normalized relevance for each hidden neuron accumulated across all three activity classes. Each bar represents how much that unit contributed overall, regardless of the specific class. A consistent color map is used for all NNs, allowing for direct visual comparison.

In the FIRNN, five neurons hold over half of the total relevance, with one hidden unit contributing 14% alone. In LSTM, the relevance is more evenly distributed, though five neurons still hold higher relevance values. GRU shows the most balanced spread, with seven neurons holding the highest relevance values, suggesting that GRU relies on a slightly broader set of internal units.

**Figure 8:**
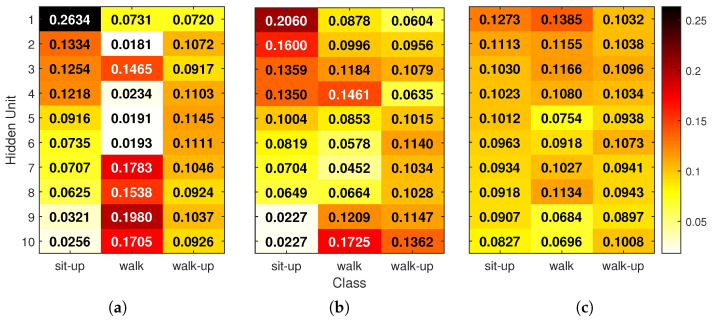
Global relevance scores for hidden units in (**a**) FIRNN, (**b**) LSTM, and (**c**) GRU. Each heat map shows the $\ell_{1}$-normalized contribution of each hidden neuron for the classification of sit-up, walking, and stair-climbing activities. The values indicate per-class ratios (columns sum to 1), and rows are sorted by decreasing relevance for the sit-up class.

To gain more detailed insight, Figure 8 shows how hidden-unit relevance is distributed per activity class. For each correctly classified test sample, absolute relevance scores at each hidden unit and time step were summed and $\ell_{1}$-normalized so that all units contributed to a total of 1. These vectors were averaged per class, resulting in a 10×3 matrix for each NN. Each heat map shows these class-wise normalized values, with numeric ratios overlaid on the cells. Rows (hidden units) are sorted by their importance for the sit-up class to help with comparison across models.

The final class-specific hidden relevance maps (Figure 8) illustrate how each neural network distributes attention across hidden units depending on the activity. In the FIRNN, a single neuron accounts for 26% of relevance for the sit-up class, indicating a highly concentrated attribution. LSTM similarly exhibits a dominant unit contributing 20% for sit-ups. For the walking class, FIRNN and LSTM, again, show pronounced peaks, with relevance concentrated at 19% and 17% in single units respectively. In contrast, the walk-up class yields a more balanced distribution: the FIRNN’s most relevant unit contributes 11%, while LSTM has two neurons above 13%.

Minimum relevance values further highlight these differences in spread. For FIRNN, the least-contributing neurons receive just 2%, 1%, and 7% relevance for sit-up, walking, and stair climbing, respectively. LSTM shows slightly more balanced lower bounds at 2%, 4%, and 6%. GRU, however, presents the most uniform distribution across all activities. For sit-up, walking, and stair climbing, hidden-unit relevance ranges between 8–12%, 6–13%, and 8–10%, respectively. This even allocation suggests that GRU’s internal representation is more robust: if any individual neuron were compromised, its impact on the final decision would likely be smaller due to the distributed nature of relevance across the hidden layer.

## 5. Discussion

This study provides a comprehensive comparison of FIRNN, GRU, and LSTM for HAR using wearable sensor data, focusing on both classification performance and explainability. The results confirm that dynamic NNs with memory capabilities, especially LSTM and GRU, can accurately capture temporal patterns in raw inertial signals. LSTM achieved the highest accuracy but at the highest computational cost, consistent with previous findings that long-term dependencies are crucial [6].

Our evaluation of computational complexity reveals that FIRNNs are significantly more efficient, making them better suited for low-power or embedded applications where resource constraints dominate. However, their lower performance on dynamic activities, particularly walking and stair climbing, suggests that recurrent connections are essential for capturing fine-grained differences between similar movement patterns.

Explainability analysis using LRP demonstrated strong agreement across models regarding sensor importance. In particular, the gyroscope Y-axis consistently emerged as the most informative input channel, while the accelerometer Y-axis contributed the least. This finding is in line with prior studies that have emphasized the role of angular velocity in recognizing repetitive full-body movements [15].

To ensure stable and interpretable LRP outputs, only models achieving the highest test accuracy within each architecture group were selected for analysis—out of more than 16,500 trained candidates. This filtering ensured that relevance maps reflect robust and converged decision patterns. Still, LRP results may vary depending on network properties such as activation functions, hidden-layer dimensionality, or learned feature sparsity. Additionally, each architecture used a distinct LRP implementation tailored to its internal structure, following established formulations for FIRNN, GRU, and LSTM models. These rule-based propagation schemes were fixed and deterministic in our case, but future work could examine how architectural variants or alternative propagation strategies affect attribution outcomes.

Additionally, all evaluations were performed on a single dataset collected in a consistent acquisition setting, using one type of sensor device and fixed sensor placement. While this controlled setup ensured fair comparisons across network architectures, it limits conclusions about model generalizability to other HAR datasets. Future work could extend this analysis by validating trained models across multiple benchmark datasets that differ in sensor type, sampling frequency, or population demographics. Such cross-dataset evaluation would help assess how architectural choices impact robustness to data heterogeneity, which is critical for real-world HAR deployment.

GRUs exhibited sharp attribution patterns, focusing on a high-relevance input channels and distributing hidden-layer relevance broadly across neurons. This contrasts with the FIRNN, where both input and hidden-layer relevance was concentrated on fewer elements. These architectural differences influence not only performance but also fault tolerance and interpretability. For example, the more distributed relevance in GRU suggests greater robustness to signal dropout, which is beneficial in noisy or mobile environments.

Additionally, our quadrant-based analysis of input relevance vs. signal variability (ZCR) highlighted that NNs prioritize highly dynamic channels for activities like walking and stair climbing, while sit-ups were recognized using less temporally variable signals. This demonstrates that neural attention adapts to both the architecture and activity type—a critical insight for designing sensor placements and neural network architectures for multi-activity HAR systems.

The hidden-layer relevance maps further revealed that FIRNN and LSTM depend on a small subset of dominant neurons per class, whereas GRU distributes relevance more uniformly. This broader engagement may reflect GRU’s internal resilience and greater redundancy, which could be useful in safety-critical or healthcare monitoring applications.

Future work may expand on these insights by applying temporal LRP variants or integrating attention mechanisms into FIRNNs to improve focus on discriminative segments. It may also be valuable to explore sensor fusion or semi-supervised learning techniques for settings with limited labeled data.

This study was conducted in a controlled environment using within-subject partitioning to ensure stable comparisons between neural network architectures. While this setup enables isolation of architectural effects on performance and explainability, it does not directly assess generalization to unseen subjects or demographic groups. The dataset itself includes a gender imbalance (75 males and 15 females), which was not specifically analyzed in this work. Although our conclusions remain valid under the examined conditions, we recognize that broader validation—including cross-subject evaluation and demographically balanced datasets—would be important for assessing model robustness in real-world deployment scenarios.

## 6. Conclusions

This work systematically compared three dynamic NN architectures—FIRNN, GRU, and LSTM—for sensor-based HAR in terms of accuracy, computational complexity, and interpretability. The key conclusions are outlined as follows:

- In the tested setting, LSTM achieved the highest accuracy (98.76%) but at a high computational cost. GRU offered a balanced compromise (97.3% accuracy), while FIRNN was most efficient but slightly less accurate (95.74%).
- LRP revealed that the gyroscope Y-axis (*gyr Y*) was the most informative input, while the accelerometer Y-axis (*acc Y*) was the least relevant across all considered NN models.
- FIRNN used a broader temporal window more uniformly, while LSTM and GRU focused on short, informative signal segments. GRU concentrated relevance into fewer sensor channels—often just one or two.
- All considered NNs aligned their attention with high zero-crossing rate inputs for dynamic activities, indicating sensitivity to temporal signal variation. Sit-up recognition relied more on stable inputs.
- FIRNN and LSTM assigned most relevance to a small number of dominant hidden neurons, while GRU distributed relevance more broadly across the hidden layer—suggesting increased resilience in scenarios involving hardware noise or neuron dropout.

These findings offer practical guidance for selecting NN architectures depending on resource constraints and application goals while improving model transparency for clinical or embedded HAR systems.

## Figures and Tables

**Table 1:** Computational complexity of FIRNN, GRU, and LSTM.

Network	$N_{2\Sigma }$	$N_{2\Pi }$	$N_{\Psi }$	$N_{W}$
FIRNN	$N_{I}N_{D}+N_{I}N_{H}+N_{H}$	$N_{I}N_{D}+N_{I}N_{H}+N_{H}$	$N_{H}+1$	$N_{H}\left(N_{I}N_{D}+1\right)+N_{C}\left(N_{H}+1\right)$
GRU	$3N_{H}N_{D}\left(N_{I}+N_{D}\right)+N_{D}$	$3N_{I}N_{H}\left(N_{I}+N_{D}N_{H}\right)$	$4N_{D}N_{H}+1$	$3N_{H}\left(N_{I}+N_{H}+1\right)+N_{C}\left(N_{H}+1\right)$
LSTM	$4N_{H}N_{D}\left(N_{I}+N_{D}\right)+N_{D}$	$4N_{I}N_{D}\left(N_{I}+N_{D}N_{H}\right)$	$6N_{D}N_{H}+1$	$4N_{H}\left(N_{I}+N_{H}+1\right)+N_{C}\left(N_{H}+1\right)$

$N_{2\Sigma }$—binary additions; $N_{2\Pi }$—binary multiplications; $N_{\Psi }$—activation functions; $N_{W}$—weights; $N_{I}$—inputs; $N_{H}$—hidden neurons; $N_{D}$—delays; $N_{C}$—output classes.

**Table 2:** Computational complexity of FIRNN, GRU, and LSTM models that achieved the highest accuracy.

Network	$N_{2\Sigma }$	$N_{2\Pi }$	$N_{\Psi }$	$N_{W}$	$N_{D}$	ACC (%)
FIRNN	670	670	11	6043	100	95.74
GRU	318,100	181,080	4001	543	100	97.33
LSTM	424,100	241,440	6001	713	100	98.76

$N_{2\Sigma }$—binary additions; $N_{2\Pi }$—binary multiplications; $N_{\Psi }$—activation functions; $N_{W}$—weights; $N_{D}$—delays.

**Table 3:** Number of input channels per activity class falling into each quadrant of the ZCR relevance count space.

Class	Network	Q1	Q2	Q3	Q4
Sit-up	FIRNN	0	2	2	2
	LSTM	0	2	2	2
	GRU	0	2	1	3
Walking	FIRNN	1	5	0	0
	LSTM	2	4	0	0
	GRU	0	6	0	0
Stair climbing	FIRNN	0	4	1	1
	LSTM	0	4	1	1
	GRU	0	4	1	1

Q1—high R and high ZCR; Q2—Low R and high ZCR; Q3—High R and low ZCR; Q4—Low R and low ZCR. R—number of relevant samples; ZCR—zero-crossing rate, in Hz.

## Data Availability

The data used in this study can be made available upon request to the corresponding author.

## Abbreviations

The following abbreviations are used in this manuscript:

ACC	Accelerometer
CAM	Class Activation Map
CNN	Convolutional Neural Network
FIRNN	Finite Impulse Response Neural Network
GRU	Gated Recurrent Unit
Grad-CAM	Gradient-weighted Class Activation Map
HAR	Human Activity Recognition
LRP	Layer-wise Relevance Propagation
LSTM	Long Short-Term Memory
NN	Neural Network
SHAP	SHapley Additive exPlanations
XAI	Explainable Artificial Intelligence
Z-score	Standard Score Normalization
ZCR	Zero-Crossing Rate
